# Effect of introduction of chondroitin sulfate into polymer-peptide conjugate responding to intracellular signals

**DOI:** 10.1186/1556-276X-6-532

**Published:** 2011-09-30

**Authors:** Tetsuro Tomiyama, Riki Toita, Jeong-Hun Kang, Haruka Koga, Shujiro Shiosaki, Takeshi Mori, Takuro Niidome, Yoshiki Katayama

**Affiliations:** 1 Graduate School of Systems Life Sciences, Kyushu University, Fukuoka 819-0395, Japan; 2 Department of Biomedical Engineering, National Cerebral and Cardiovascular Center Research Institute, 5-7-1 Fujishiro-dai, Suita, Osaka 565-8565, Japan; 3 Department of Applied Chemistry, Faculty of Engineering, Kyushu University, 744 Motooka, Nishi-Ku, Fukuoka 819-0395, Japan; 4 Center for Future Chemistry, Kyushu University, 744 Motooka, Nishi-Ku, Fukuoka 819-0395, Japan

## Abstract

We recently developed a novel tumor-targeted gene delivery system responding to hyperactivated intracellular signals. Polymeric carrier for gene delivery consists of hydrophilic neutral polymer as main chains and cationic peptide substrate for target enzyme as side chains, and was named polymer-peptide conjugate (PPC). Introduction of chondroitin sulfate (CS), which induces receptor-medicated endocytosis, into polymers mainly with a high cationic charge density such as polyethylenimine can increase tumor-targeted gene delivery. In the present study, we examined whether introduction of CS into PPC containing five cationic amino acids can increase gene expression in tumor cells. Size and zeta potential of plasmid DNA (pDNA)/PPC/CS complex were <200 nm and between -10 and -15 mV, respectively. In tumor cell experiments, pDNA/PPC/CS complex showed lower stability and gene regulation, compared with that of pDNA/PPC. Moreover, no difference in gene expression was identified between positive and negative polymer. These results were caused by fast disintegration of pDNA/PPC/CS complexes in the presence of serum. Thus, we suggest that introduction of negatively charged CS into polymers with a low charge density may lead to low stability and gene regulation of complexes.

## Introduction

Tumor-targeted gene delivery is beneficial for increasing therapeutic effects and reducing the desired side effect. These gene delivery systems are mainly based on passive targeting and active targeting methods. Nanoparticles pass through leaky, tortuous, and heterogeneous angiogenic tumor blood vessels and tend to accumulate more in tumors than in normal tissues showing a well-organized and functional structure. This phenomenon is known as the enhanced permeability and retention effect and is noteworthy as the passive targeting method. Moreover, tumor cells exhibit several hyperactivated receptors and cellular signals (protein kinases and proteases) which play key roles on tumor growth and survival and angiogenesis. Gene delivery systems recognizing these hyperactivated receptors or cellular signals are the main active targeting methods [1].

Recently, our group has proposed a novel drug or gene delivery system responding to cellular signals and developed some gene regulation systems corresponding to the target protein kinases or proteases such as protein kinase A [2-4], caspase [5], Ikappa-B kinase [6], human immunodeficiency virus-1 protease [7], and protein kinase C [8-12]. Carriers for delivery of genes consisted of neutral and hydrophilic polymer as the main chain and disease-specific peptide substrate as side chains; we named polymer-peptide conjugates (PPC). The disease-specific peptide substrates containing positive amino acids can bind anionic genes. Phosphorylation by protein kinases or cleavage by proteases leads to disintegration of PPC/gene complexes and gene expression. Gene delivery systems recognizing hyperactivated cellular signals show efficient disease-targeted gene delivery.

Chondroitin sulfate (CS) is a negatively charged glycosaminoglycan containing two alternating monosaccharides (*N*-acetylgalactosamine and glucuronic acid). It recognizes a CD44 receptor, which is hyperactivated on several tumor cells [13-15], leading to receptor-medicated endocytosis.

In the present study, we examined whether introduction of CS into pDNA/PPC complex can increase gene expression in tumor cells. Diameter, zeta potential, and stability of plasmid DNA (pDNA)/PPC/CS complexes and their transfection efficiency for tumor cells were elevated.

## Results and discussion

PPC consisted of polyacrylamide as the main chain and the peptide substrate as side chains. The content of peptide as the side chains of the polymer was estimated to be 2.9 mol% for the positive polymer [PPC(S)] containing the phosphorylation site serine (FKKQG***S***FAKKK) and 2.8 mol% for the control negative polymer [PPC(A)] that the serine in the peptide was substituted with alanine (FKKQG***A***FAKKK) by using an elemental analysis, respectively. Since the peptide substrate has five cationic amino acids (lysine), it can bind to anionic pDNA. After binding PPC with pDNA, CS was introduced into pDNA/PPC complex.

**Figure 1:**
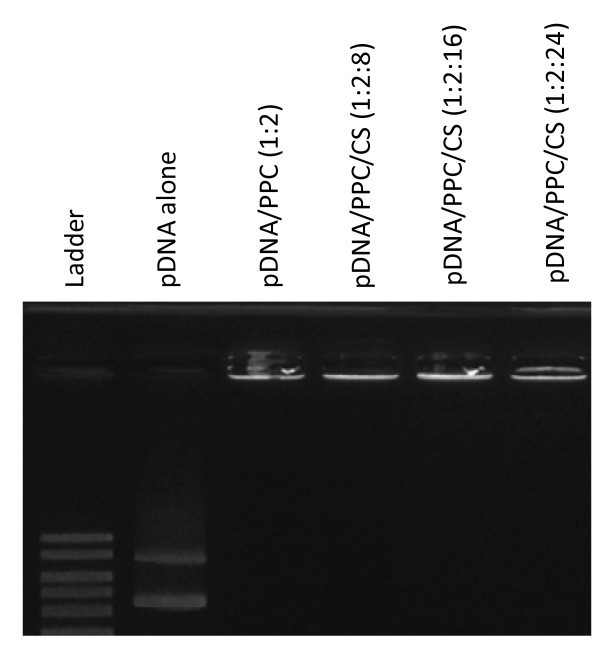
**Electrophoresis of pDNA alone, pDNA/PPC, or pDNA/PPC/CS complexes**. Various concentrations of polymer were mixed with the pDNA and analyzed by 1% agarose gel electrophoresis.

We investigated whether PPC or PPC/CS can form a stable complex with pDNA. When polymers were added to the pDNA solution, the migration of pDNA was suppressed during gel electrophoresis (Figure 1). This result means that PPC or PPC/CS formed a stable complex with pDNA through electrostatic interaction.

**Figure 2:**
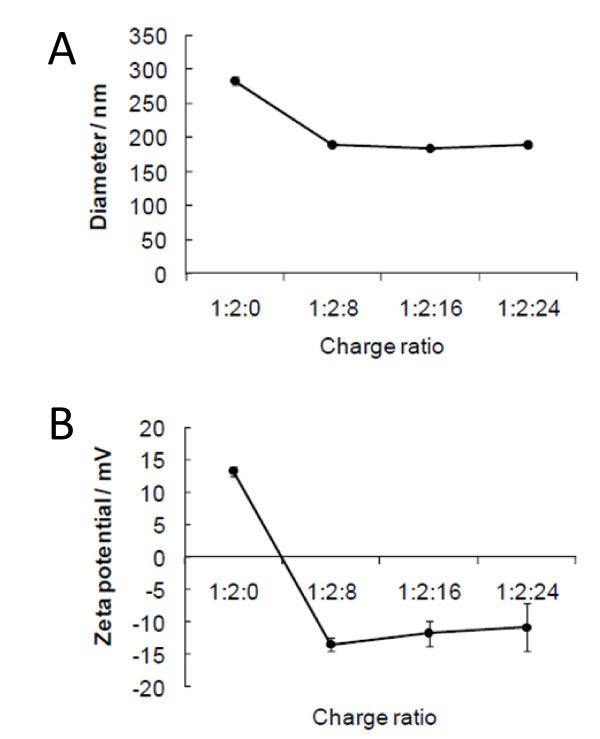
**(A) Diameter and (B) zeta potential of complexes**. After binding PPC with pDNA, CS was added into pDNA/PPC complex in water. Diameters and zeta potentials of the complexes were determined using a Zetasizer.

To find optical CS concentration, we determined zeta potentials for several charge ratios of pDNA/PPC/CS. Complexes became a plateau at a charge ratio 1:2:16 of pDNA/PPC/CS, and their size and zeta potential were 178 nm and -12 mV, respectively (Figure 2). In this study, we used pDNA/PPC/CS complex at a charge ratio of 1:2:16.

Staining with WST-8 was used to identify cytotoxicity of pDNA/PPC (charge ratio = 1:2) or pDNA/PPC/CS (charge ratio = 1:2:16) complexes toward HepG2 cells. Twenty-four hours after incubation with complexes, the percentage of viable cells was calculated. The assay results revealed that the complexes did not affect cell viability (>90%) (data not shown).

**Figure 3:**
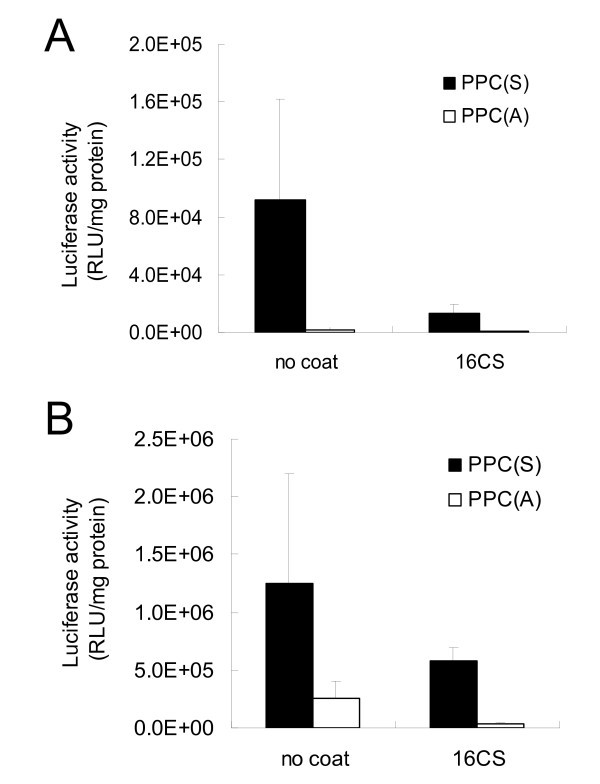
**Luciferase expression after transfection of complexes**. Complexes were transfected into (A) HepG2 and (B) B16 melanoma cells and luciferase expression was detected at 24 h after transfection. The results are presented as the RLU per milligram of total protein. Error bars represent standard deviation of three experiments.

**Figure 4:**
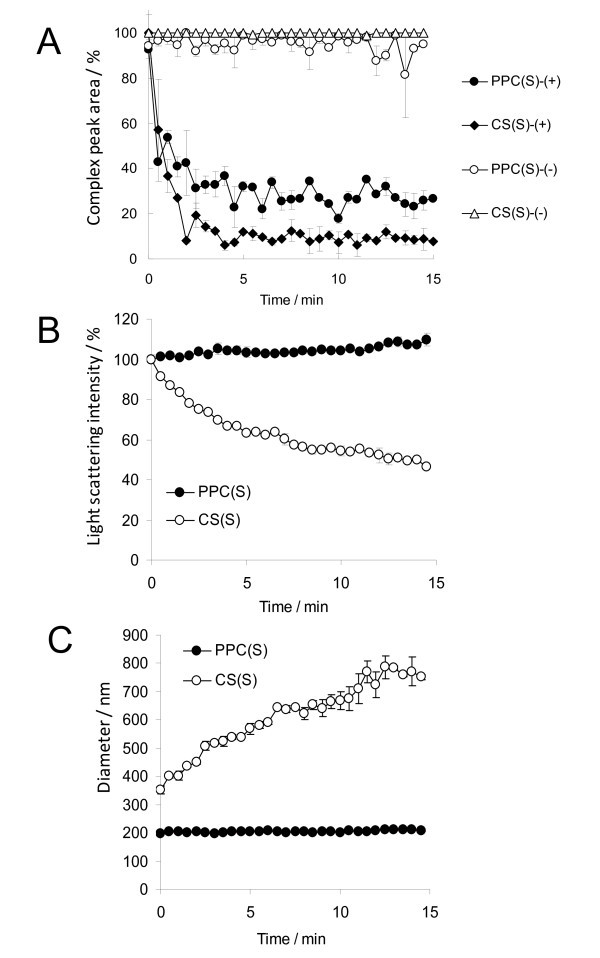
**(A) Change of complex peak area in 10% FBS**. Change of light scattering intensity (LSI) (B) and (C) diameter of complex in Opti-MEM medium. Peak area, diameter, and zeta potential of the complexes were determined using a Zetasizer.

To examine whether introduction of CS into pDNA/PPC complex can increase gene expression in tumor cells, pDNA/PPC (charge ratio = 1:2) and pDNA/PPC/CS (charge ratio = 1:2:16) complexes were transfected into B16 melanoma and HepG2 cells. The pDNA/PPC/CS complex showed lower gene expression in both tumor cells, compared with that of pDNA/PPC complex (Figure 3). In previous studies, for receptor-mediated gene delivery, CS was introduced polymers mainly with a high cationic charge density such as polyethylenimine (PEI) [16,17]. On the other hand, PPC carrier contains five cationic amino acids, meaning that introduction of CS into pDNA/PPC complex may make complexes weaken. Thus, we investigated stability of pDNA/PPC and pDNA/PPC/CS complexes in the presence of Opti-MEM or 10% fetal bovine serum (FBS). The pDNA/PPC complex showed stable diameters and light scattering intensity in Opti-MEM, in the case of pDNA/PPC/CS complex, but their time-dependent changes were observed (Figure 4). In the presence of 10% FBS, both pDNA/PPC and pDNA/PPC/CS complexes were disintegrated, but the speed of disintegration was rapider in pDNA/PPC/CS than pDNA/PPC complexes. These results indicate that introduction of CS into pDNA/PPC complex makes the complex weak and unstable.

Cationic polymers with a high cationic charge density form stable complexes with gene and show high transfection efficiency but have high cytotoxicity toward cells and agglutination with blood components. Introduction of CS into these polymers can reduce cytotoxicity and agglutination and lead to receptor-mediated gene delivery [16,17]. However, there are very few evaluation studies for introduction of CS into polymers with a low cationic charge density. Our study suggest that introduction of negatively charged CS into polymers with a low charge density leads to low stability and gene regulation of complexes.

## Materials and methods

### pDNA/PPC/CS complex

PPC was synthesized as described previously [9]. Briefly, acrylamide (41.2 mg, 581 μmol) and *N*-methacryloylpeptide (8.0 mg, 5.9 μmol), from which the methacryloyl group attached to the amino terminus of the peptide, were dissolved in water, degassed with nitrogen for 5 min, and then polymerized using ammonium persulfate (3.9 mg, 17 mmol) and *N*,*N*,*N*',*N*',-tetramethylethylenediamine (5.1 μl, 34 mmol) as a redox couple at room temperature for 90 min. The synthesized sample was dialyzed against water overnight in a semi-permeable membrane bag with a molecular weight cutoff of 50,000. The dialyzed sample was lyophilized, and a final sample was obtained as a white powder, which was used as the polymer for PPC. The concentration of the peptide was estimated by elemental analysis.

For making pDNA/PPC/CS complex, 20 μl of pDNA (pCMV-luciferase) (0.1 μg/μl) and 20 μl of PPC (640 μM) were mixed for 20 min at room temperature and then incubated further for 20 min after addition of CS (3.98 mM) into pDNA/PPC solution.

### Measurements of zeta potential and diameter of complexes

pDNA/PPC/CS complex solution at a charge ratio of 1:2:16 was adjusted to 1 ml by 10 mM HEPES buffer (pH 7.3) or Opti-MEM (Gibco, Invitrogen Co., Grand Island, NY, USA). The zeta potential and diameter of the complexes were determined using a Zetasizer (Malvern Instruments, London, UK) with the He/Ne laser (633 nm) at a detection angle of 173° and a temperature of 25°C.

### Agarose gel electrophoresis

pDNA (0.1 μg) and polymers were mixed in 10 μl HEPES buffer (10 mM) at several charge ratios. The formulation of complex was assayed by 1% agarose gel electrophoresis.

### Cytotoxicity of complex toward cells

Cell viability was determined using a cell counting kit containing 4-[3-(2-methoxy-4-nitrophenyl)-2-(4-nitrophenyl)-2H-5-tetrazolio]-1,3-benzene disulfonate sodium salt (WST-8) (Dojindo Laboratories, Kumamoto, Japan). HepG2 cells were incubated in the absence or presence of pDNA/PPC (charge ratio = 1:2) or pDNA/PPC/CS complex (charge ratio = 1:2:16) for 24 h in a 96-well plate. The conditioned medium in each well was replaced with 100 μL of fresh medium containing WST-8, and the cells were incubated for a further 2 h, before measurement of the absorbance at 440 nm. The cell viability (percent) was calculated by normalizing the absorbance of treated cells to that of the untreated control cells.

### Transfection of complexes into cells

Cells (5 × 10^4^) were grown in Dulbecco's modified Eagle's medium (DMEM; Gibco Invitrogen Co.) supplemented with 10% fetal bovine serum (FBS), 100 U/ml penicillin, 100 μg/ml streptomycin, and 0.25 μg/ml amphotericin B (all Gibco). The cells were incubated in a humidified atmosphere containing 5% CO_2 _and 95% air at 37°C for 24 h. After 24 h, the medium was changed to 500 μL of Opti-MEM (Gibco, Invitrogen Co.), and pDNA/PPC (charge ratio = 1:2) and pDNA/PPC/CS (charge ratio = 1:2:16) complexes were added to wells; concentration of pDNA was 2 μg/well. The cells were incubated at 37°C for 6 h. After 6 h, the medium was changed to DMEM, and cells were further incubated for 18 h. The cultured cells were then scraped and lysed in 100 μL of lysis buffer (20 mM Tris-HCl, pH 7.4, 0.05% Triton-X 100, and 2 mM EDTA). After centrifuging the sample at 12,000 × *g *at 4°C for 10 min, a 10 μL aliquot of the supernatant was used for measuring chemiluminescence in a MiniLumat LB 9506 (EG & G Berthold, Wildbad, Germany) directly after adding 40 μL of the luciferin substrate. The results are presented as relative luminescence units (RLU) per milligram of total protein.

## Competing interests

The authors declare that they have no competing interests.

## Authors' contributions

TT, RT, and YK designed the experiments. TT, RT, JHK, HK, SS, TM, and TN performed the experiments and analyzed the data. JHK and YK wrote the manuscript.
